# Using homemade stainless steel dendrometer band for long term tree growth measurements

**DOI:** 10.1186/s40529-023-00395-8

**Published:** 2023-07-19

**Authors:** Chih-Hsin Cheng, Pei-Chen Lee, Hong-Rue Lee, Chiou-Pin Chen, Oleg V. Menyailo

**Affiliations:** 1 School of Forestry and Resource Conservation, National Taiwan University, Taipei, 106 Taiwan; 2 NTU Experimental Forest, National Taiwan University, Taipei, 106 Taiwan; 3 Joint FAO/IAEA Centre of Nuclear Techniques in Food and Agriculture, Soil and Water Management & Crop Nutrition Laboratory, Seibersdorf, 2444 Austria

**Keywords:** Diameter tape, Diameter increment, Tree growth, Diameter at breast height (DBH), Basal area

## Abstract

Dendrometer bands have been proposed as an accurate method for measuring tree growth. However, the constrained observation window and the material used in them hamper long-term tree growth monitoring. This study devised a dendrometer band made from stainless steel and primarily extended the extension length of the band spring to yield ample space to monitor diameter increments long-term. A total of more than 500 individual trees, including both coniferous and broadleaf trees, were examined. We compared the dendrometer band’s long-term performance with diameter tape for 5- and 10-year measurements. The results showed that the measurements of the two methods were highly correlated (R > 0.89) in both measuring periods. Differences between the two measurements for individual trees were typically less than 5 mm, and the mean differences at a stand level were less than 2 mm. These consistent observations suggested that the dendrometer band measurements were reliable for long-term measurement. Using the dendrometer bands, we further demonstrated the annual tree growths of diameter at breast height (DBH) and basal area (BA) for ten years of measurements. The size-dependent relationships between DBH/BA growth and initial DBH were also presented. Owing to their simple installation, low cost, and reliable measurement, these dendrometer bands would be helpful in forestry and forest ecology research.

**Supplementary Information:**

The online version contains supplementary material available at 10.1186/s40529-023-00395-8.

## Introduction

Measuring tree growth is a vital part of ecological and silvicultural studies of forests. Tree growth measurements provide information about forest health, productivity, sustainability, and the potential for carbon sequestration, which is critical in mitigating greenhouse gas emissions (Pan et al. 2011). Researchers commonly measure tree growth by evaluating diameter increments recorded at successive inventories (Biondi 1999). By integrating the growth of individual trees under different measuring periods, the stand growth can be quantified and represent the overall growth condition of the forest.

The typical method for measuring diameter increments involves using calipers or diameter tape at breast height at fixed time intervals. As sufficient time must elapse between measurements to achieve a confident estimate, these measurements are less useful as short-term indicators and are usually collected at 5-to-10-year intervals (Biondi 1999; Schliep et al. 2014). Interpolation is required for computing the yearly growth. Moreover, failure to measure at the same height or on the proper plane may introduce error and reduce accuracy (Weaver et al. 2015). Negative diameter increments are occasionally observed despite annual trees growth (Clark and Clark 1999).

Dendrometer bands have been developed by researchers and are used to make repeated measurements of tree growth (Breitsprecher and Hughes 1975; Cattelino et al. 1986; Muller-Landau and Dong 2010; Carvalho and Fefili 2011; Anemeat and Middleton 2013). The bands have been successfully used for hourly (Raffelsbauser et al. 2019), daily (Deslauriers et al. 2007), weekly (Bormann and Kozlowski 1962), monthly (Pelissier and Pascal 2000), and annual diameter increment measurements (Keeland and Sharitz 1993). Most dendrometer bands perform well after a few years in the field (Pesonen et al. 2004; Drew and Downes 2009). However, the materials used in them or the short observation window of the dendrometer bands hamper the use of these bands for monitoring tree growth for extended periods (Cattelino et al. 1986; Muller-Landau and Dong 2010). To maintain accurate monitoring, old dendrometer bands must be replaced periodically. Extending the service time of the dendrometer bands would thus enable more convenient measurements.

In this study, we devised a dendrometer band made from stainless steel and primarily extended the maximum extension length of the band spring at 44 cm to yield ample space to monitor diameter increments long-term. A total of more than 500 individual trees, including both coniferous and broadleaf trees, were examined. We compared the dendrometer band’s long-term performance with diameter tape for 5- and 10-year measurements. Our objectives were to investigate the reliability of long-term field measurements made with the dendrometer bands and highlight their potential use in forest ecology and management applications.

## Materials and methods

### Study sites

The study was conducted in the National Taiwan University Experimental Forest in Xitou, Taiwan (23°40’N, 120°47’E). The mean annual temperature over the last 30 years in Xitou was 16.6°C, and the mean annual precipitation was 2635 mm. Typhoons and heavy rainstorm events are common natural disturbances in summer and fall in the area (Cheng et al. 2020).

A total of nine stands with stand ages ranging from 14 to 92 years were selected in this study. The selected stands were classified as coniferous plantations (CP) and broadleaf plantations (BR). The oldest stands, from CP1 to CP4, were Japanese cedar (*Cryptomeria japonica*), and the middle-aged stands, from CP5 to CP7, were Chinese fir (*Cunninghamia lanceolata*) and *T. cryptomerioides*. The two youngest BR stands were *Cerasus campanulate* and *Acer serrulatum* at BR1 and *Liquidambar formosana* at BR2. The mean diameter at breast height (DBH) of the stands ranged from 12.6 cm at BR2 to 48.0 cm at CP1 and increased with stand age (Table 1) (Cheng et al. 2020).

**Table 1:** Stand characteristics of the selected coniferous (CP) and broadleaf (BR) stands in Xitou, central Taiwan

Site	Species	Year planted	Altitude	Tree density	Mean DBH^*a*^	BA^*b*^	Canopy height	Dendrometer bands
			(m)	(no. ha^− 1^)	(cm)	(m^2^ ha^− 1^)	(m)	(no.)
CP1	*Cryptomeria japonica*	1920	1050	408	48.6	77.9	30.9	30
CP2	*Cryptomeria japonica*	1960	1170	658	33.9	64.0	27.6	52
CP3	*Cryptomeria japonica*	1973	1370	1358	23.1	59.7	20.8	67
CP4	*Cryptomeria japonica*	1971	1300	806	34.9	80.1	23.5	74
CP5	*Cunninghamia lanceolata*	1979	1100	762	29.5	53.7	24.1	76
CP6	*Taiwania cryptomerioides; Cunninghamia lanceolata*	1981	1100	654	29.4	47.2	24.3	93
CP7	*Taiwania cryptomerioides*	1988	1270	1068	23.9	49.3	18.7	83
BR1	*Cerasus campanulate;* *Acer serrulatum*	1988	1200	616	12.6	11.5	7.2	19
BR2	*Liquidambar formosana*	1998	950	988	13.2	13.9	11.1	23

^*a*^DBH: diameter at breast height

^*b*^BA: basal area

### Dendrometer bands

**Fig. 1:**
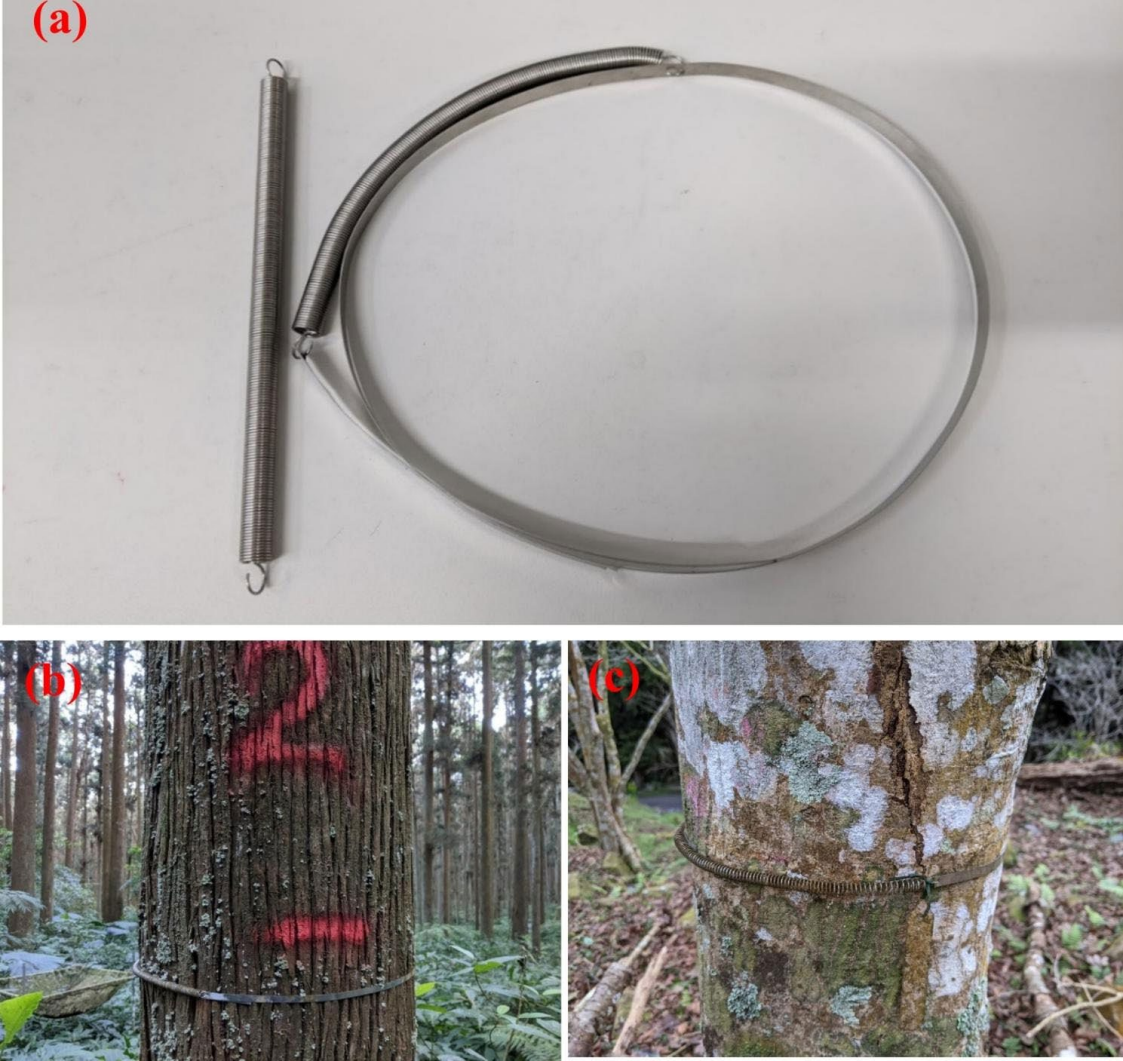
(**a**) Stainless steel band and spring used in this study. (**b**), (**c**) Dendrometer bands wrapped on a *Japanese cedar* tree and an *Acer serrulatum* tree after 10 years.

The dendrometer bands comprised two essential elements: a steel band strap and a steel spring (Fig. 1). Both parts were made of 304 stainless steel alloy, which is strong, corrosion-resistant, and has low thermal expansion. The steel bands had a width of 8 mm and a thickness of 0.3 mm (Anemeat and Middleton 2013). The spring had a wire diameter of 0.8 mm, an outside diameter of 8 mm, a length of 150 mm, and 187 coils. Figure S1 in the Supplementary Materials presents the relationship between the extension length and force. The extension force was approximately 5 N for every 10 cm extension. The maximum extension limit of the spring was 44 cm, which gave the bands ample space to monitor diameter increments long-term. Thus, the spring can perform well for 14 years under DBH increment at 10 mm yr^− 1^.

A wire brush, metal snip, hammer, nail, and gloves were used in the field to help install the dendrometer bands (Figure S2). The wire brush was used to clean the trunk to remove moss, loose dirt, and bark that might interfere with measurements; the metal snip was used to cut the band to the desired length and make a notch at an initial position, and the hammer and concrete nail were used to punch holes in the band to attach the spring.

An example of constructing dendrometer bands is illustrated in Figure S3 and can also be referred to the previous studies (Cattelino et al. 1986; Muller-Landau and Dong 2010; Anemeat and Middleton 2013). We first punched a hole at the terminal end of the band and attached one end of the spring to the hole. We then wrapped the band on the trunk at the breast height of 1.3 m and marked the position in the middle of the band at the spring stretched at 1.5 times its length (approximately 8–10 cm of stretch). The band was loosened from the trunk, and a second hole was punched at the marked position. The band was then wrapped horizontally on the trunk again, and the other end of the spring was attached through the second hole. After the spring was connected, the band would be placed tightly on the trunk. The band was cut at approximately 15 cm extending to the spring. This “free-moving” end of the band was snugly tucked under the band and spring, and a notch was made in the band precisely at the edge of the free-moving end. The notch was used as an initial position for recording subsequent tree growth, and the free-moving end of the band moved away from the notch as the tree expanded or contracted. The scale should read 0.00 mm at installation.

The installation time for each dendrometer band was approximately 5 minutes (not including the time required for tree selection and smoothing the bark). Between 19 and 93 individual trees in each stand were selected for dendrometer band installation. A total of 517 dendrometer bands were installed at the end of 2011 (Table 2). The DBH of each tree was also measured using diameter tapes at a location on the tree trunk. The measurement position was painted with red spray paint to ensure that subsequent measurements were made at the same location. The initial DBHs of the selected trees were between 16.4 and 71.6 cm for the coniferous trees and between 7.0 and 26.3 cm for the broadleaf trees.

**Table 2:** Diameter increments (mm) measured with diameter tapes and dendrometer bands, and the differences and correlation coefficients between the two measurements for 5-year (2012–2016) and 10-year (2012–2021) measuring periods in Xitou, central Taiwan

	# Bands	Diameter tapes (mm)			Dendrometer bands (mm)			Differences (mm)			Correlation coefficient	Paired t-test
		Mean	Range		Mean	Range		Mean	Range		R	*p*-value
**2012–2016 (5 years measuring period)**												
CP1	n = 28	8.5 ± 7.6^*a*^	-1.6–23.9		8.7 ± 5.6	0–22.3		-0.2 ± 3.8	-9.7–5.3		0.87	*p* = 0.79
CP2	n = 50	9.0 ± 6.6	0–27.1		9.2 ± 6.4	0.4–25.9		-0.1 ± 1.8	-4.0–3.4		0.96	*p* = 0.53
CP3	n = 65	5.6 ± 6.1	-1.6–27.1		7.2 ± 6.0	0.3 − 24.9		-1.6 ± 2.3	-6.7–2.9		0.93	***p*** **< 0.01**
Total	n = 144	7.4 ± 6.7	-1.6–27.1		8.2 ± 6.1	0–25.9		-0.8 ± 2.6	-9.7–5.3		0.92	***p*** **< 0.01**
**2012–2021 (10 years measuring period)**												
CP1	n = 28	20.8 ± 12.8	0–49.4		19.6 ± 12.1	0–47.9		1.2 ± 4.1	-10.6–9.7		0.95	*p* = 0.12
CP2	n = 50	17.9 ± 11.0	0–47.8		16.8 ± 10.9	0.5–45.5		1.1 ± 2.6	-8.5–6.9		0.97	*p* = 0.56
CP3	n = 65	14.5 ± 15.1	-3.2–60.5		16.7 ± 14.6	0–60.5		-2.2 ± 3.0	-9.7–10.0		0.98	***p*** **< 0.01**
CP4	n = 72	26.3 ± 13.5	0–52.5		25.7 ± 12.8	1.8–51.6		0.6 ± 3.4	-9.9–8.9		0.97	*p* = 0.13
CP5	n = 69	39.4 ± 26.8	-1.6–105.1		38.5 ± 26.1	0.3–102.2		1.0 ± 3.8	-8.6–20.1		0.99	*p* = 0.12
CP6	n = 89	45.9 ± 30.4	0–116.2		46.0 ± 29.8	0.8–115.6		-0.1 ± 2.4	-4.0–6.7		0.99	*p* = 0.15
CP7	n = 80	36.0 ± 23.4	-1.6–89.2		36.1 ± 23.1	1.8–92.0		-0.2 ± 3.3	-11.8–7.2		0.99	*p* = 0.58
BR1	n = 17	60.5 ± 28.6	1.6–92.3		61.1 ± 29.5	1.4–93.0		0.7 ± 4.5	-7.0–11.8		0.99	*p* = 0.51
BR2	n = 22	50.4 ± 40.0	3.2–125.8		58.6 ± 44.7	3.0–135.4		-8.2 ± 6.4	-22.3–0.2		0.99	***p*** **< 0.01**
Total	n = 492	33.1 ± 26.4	-3.2–125.8		33.4 ± 26.7	0-135.4		-0.3 ± 3.9	-22.3–20.1		0.99	*p* = 0.16

^*a*^Mean ± standard deviation

The diameter increment of the dendrometer bands was measured at least once every year from 2012 to 2021. We conducted the measurements in winter between December and early February. We used digital calipers (± 0.01 mm precision) or cloth rulers (if the circumference increment was over 130 mm) to measure the distance between the moving end of the band and the notch (Figure S3). In 2016 (only at CP1, CP2, and CP3 stands) and in 2021, 5 and 10 years, respectively, after the installation of the dendrometer bands, we used diameter tapes to remeasure the tree diameters at the same position. We compared the tree growth data between the two measurements. The purpose of the measurements with the diameter tape was to validate the long-term durability and reliability of the dendrometer bands. We did not mean that the data measured with the diameter tapes were more accurate than those measured with the dendrometer bands. The measurement intervals between 5 and 10 years are commonly practiced in Taiwan. These more intensive measurements at CP1, CP2, and CP3 were given because the stands belonged to the project for monitoring tree growth of old-growth Japanese cedar plantations (Cheng et al. 2013).

### Data analysis

The circumference measurements from both methods were divided by π to obtain the linear diameter increments of the trees. To maximize comparison accuracy, data were discarded if a tree was dead, presumed dead, or missing from the census. The total number of assessed trees was 492. We calculated the absolute differences between two measurements and further performed correlation analysis and a paired t-test. The difference values were calculated as the diameter increments measured with diameter tape minus those measured with dendrometer bands. For the paired t-test comparison, the data were first examined for normality (*p* > 0.05) before running the test. If the data violated the normality condition, a nonparametric method, Wilcoxon’s matched-pairs signed-rank test, was used to determine whether pairs of sample sets were significantly different.

We selected CP1 and CP6 as the exemplified stands to demonstrate the applications of using bands in forests. We presented the annual DBH/basal area (BA) growths of induvial trees for ten years of measurements. The size-dependent relationships between DBH/BA growth and initial DBH were also presented. These two parameters were commonly examined in forest inventory census but undertaken for extended measuring periods such as 5 or 10 years. A finer scale resolution could examine the tree growth patterns and variations more precisely that favor better evaluation of stand development and management. However, the demonstration here is not to assess tree growth models but to illustrate the applications of bands in forests. We used SigmaPlot version 14.5 (SYSTAT, Palo Alto, California, USA) for statistical analyses.

## Results

### Homemade steel dendrometer bands

**Fig. 2:**
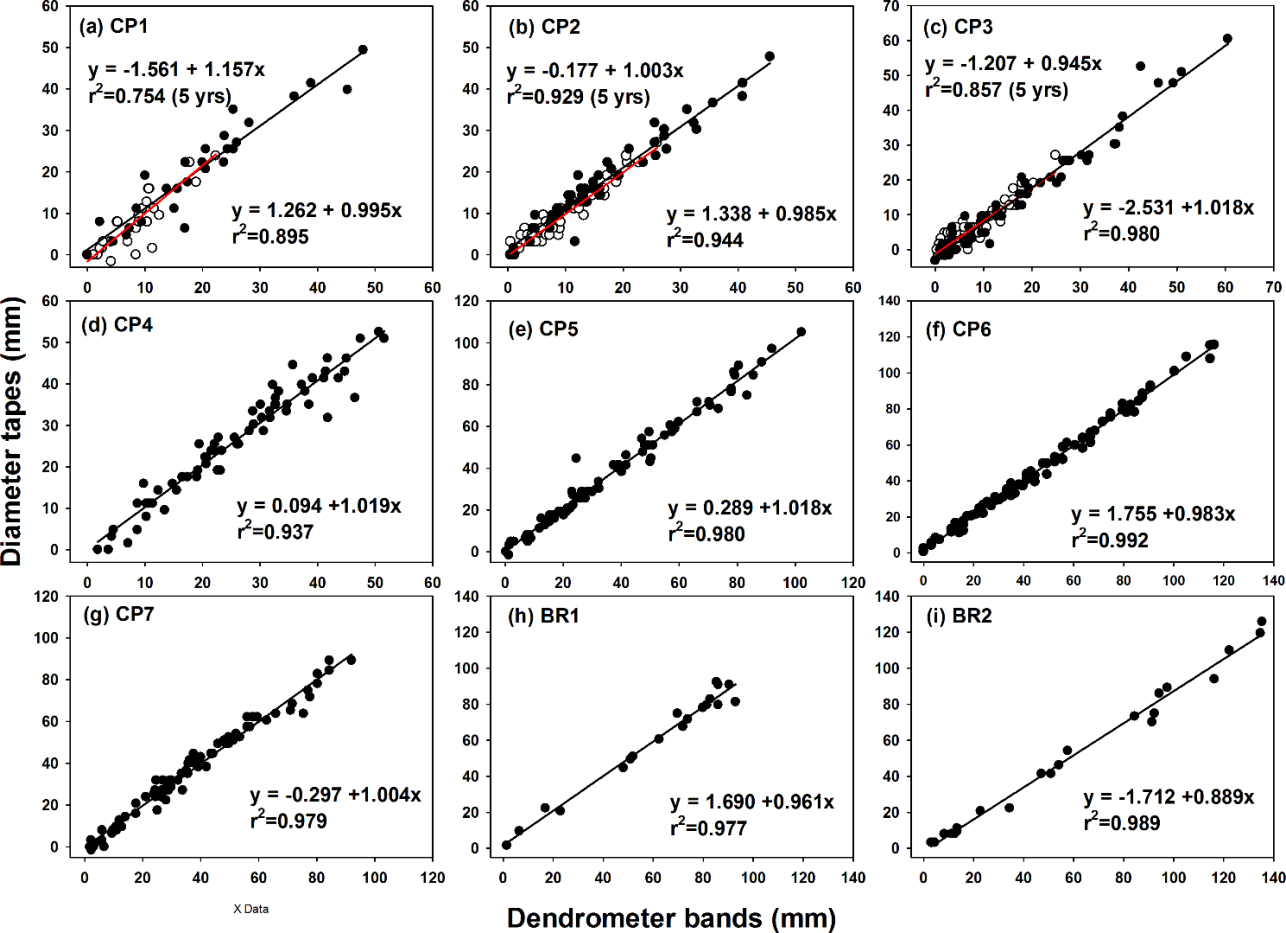
Linear relationships of diameter increments measured with diameter tapes and dendrometer bands on coniferous (from CP1 to CP7) and broadleaf (BR1 and BR2) stands in Xitou, central Taiwan. The open circles and red lines represent the data from the 5-year measurement period, and the solid circles and black lines indicate data from the 10-year measurement period.

**Fig. 3:**
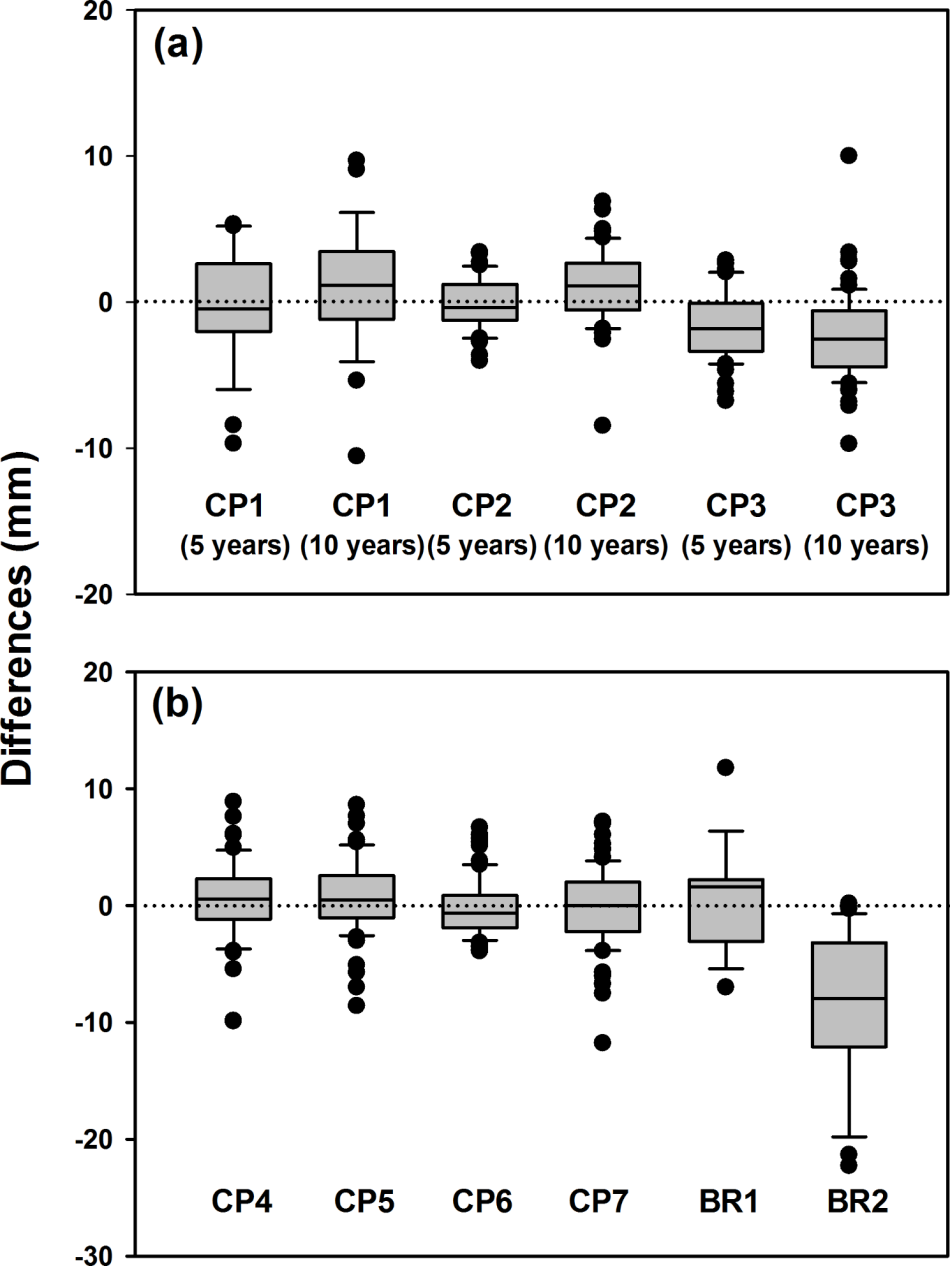
Box plots presenting differences of diameter increments measured with diameter tapes and dendrometer bands. The difference was calculated as the diameter increments measured with diameter tape minus those measured with dendrometer bands at the (**a**) CP1, CP2, and CP3 stands for the 5- and 10-year measurement periods, and (**b**) other six stands for the 10-year measurement period.

The diameter increments of the individual trees over the 5-year measurement period ranged from 1.6 to 29.1 mm with the diameter tape measurements and from 0 to 25.9 mm with the dendrometer bands measurements (Table 2). The mean diameter increments at the three stands (i.e., CP1, CP2, and CP3 which more intensive measurements were given with the project in monitoring tree growth of old-growth Japanese cedar plantations) ranged from 5.2 to 9.2 mm for both measurements. The diameter increments of the individual trees for the 10-year measuring period were between − 3.2 and 125.8 mm and between 0 and 135.4 mm for the diameter tape and dendrometer band measurements, respectively. The mean diameter increments at the nine stands ranged from 14.5 to 60.5 mm for the diameter tape measurements and from 16.7 to 61.1 mm for the dendrometer band measurements. In general, the two methods yielded consistent measurements. The differences between the two measurements of the individual trees were typically less than 5 mm (Figs. 2 & 3). Mean differences between two measurements were typically less than 2 mm; however, average differences of -8.2 mm and − 2.2 mm were observed in BR2 and CP3, respectively, for the 10-year measuring period (Fig. 3; Table 2).

The two measurements were highly correlated. The correlation coefficients were larger than 0.87 for all stands for both the 5- and 10-year measuring periods (Table 2). For all stands except for CP3 and BR2, no significant difference was observed between the measurements. The diameter increments measured with the diameter tape were lower than those measured with the dendrometer bands at CP3 and BR2. In the pooled data (n = 492 for the 10-year measuring period), no significant difference was observed between the two measurements (*p* = 0.16). Thus, based on all the band measurements, we could not reject the null hypothesis that the values were from the same population.

### Applications in forest management: examples from CP1 and CP6

**Fig. 4:**
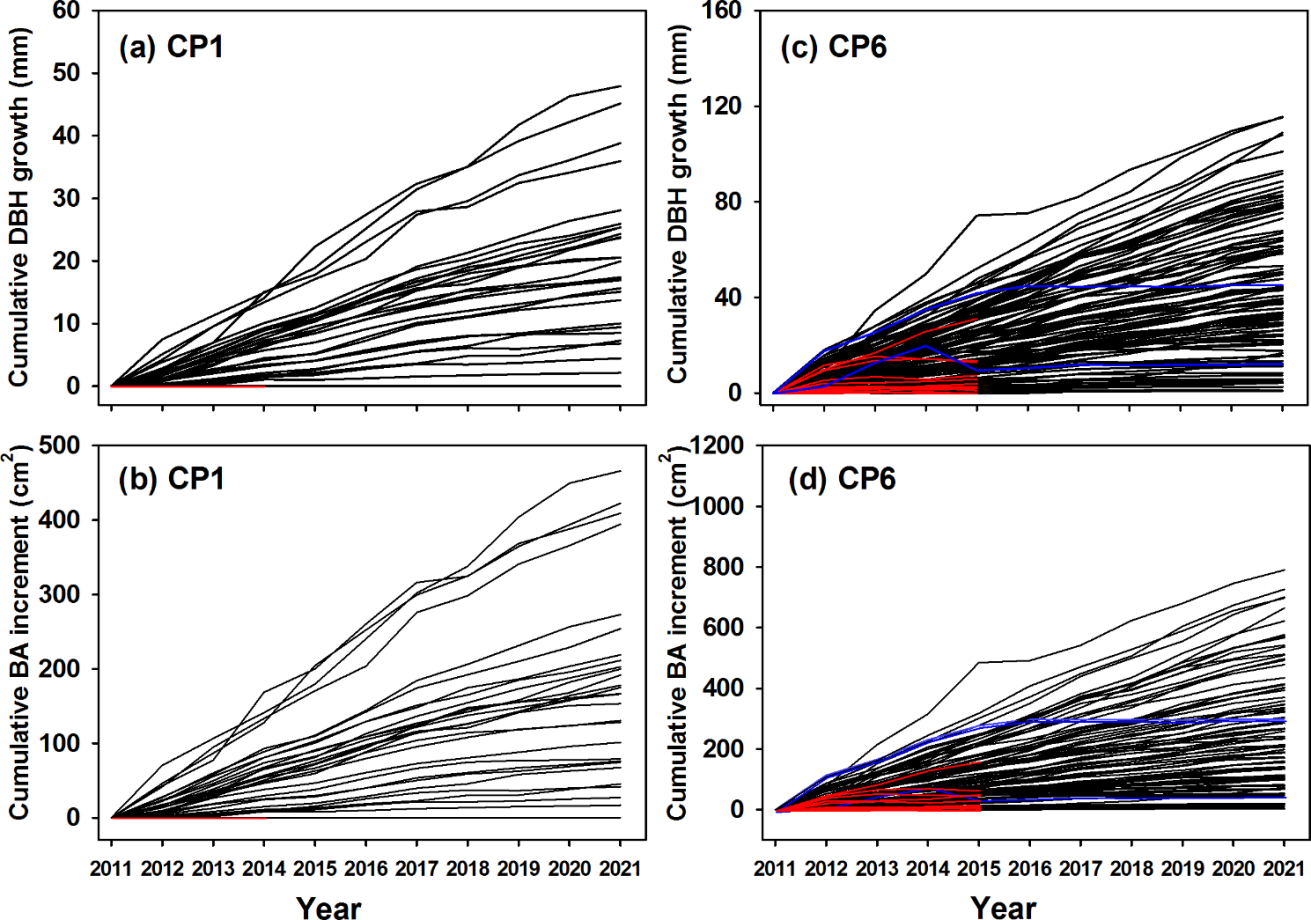
Cumulative tree DBH/BA growth of each individual tree (solid black lines) from 2012 to 2021 at CP1 (**a, b**) and CP6 (**c, d**) stands. The red lines represent the tree growth of the dead trees that died during measurement periods, and the blue lines represent the tree growth of the trees with stem breakage.

Figure 4 shows examples of cumulative tree growth measured by the dendrometer bands from 2012 to 2021 at CP1 and CP6 stands. The dendrometer bands monitored tree growth at a fine-scale resolution. It was interesting to observe that most trees grew at a similar growth pattern, in which the fast-growing trees grew faster and kept the growing paces over ten years. The irregular patterns, like the decline in diameter increment, were found in the trees with stem breakage (e.g., blue lines in Fig. 4(c) and 4(d)). The growth of the dead trees that died during measurement periods was also shown in Fig. 4 (red lines).

**Fig. 5:**
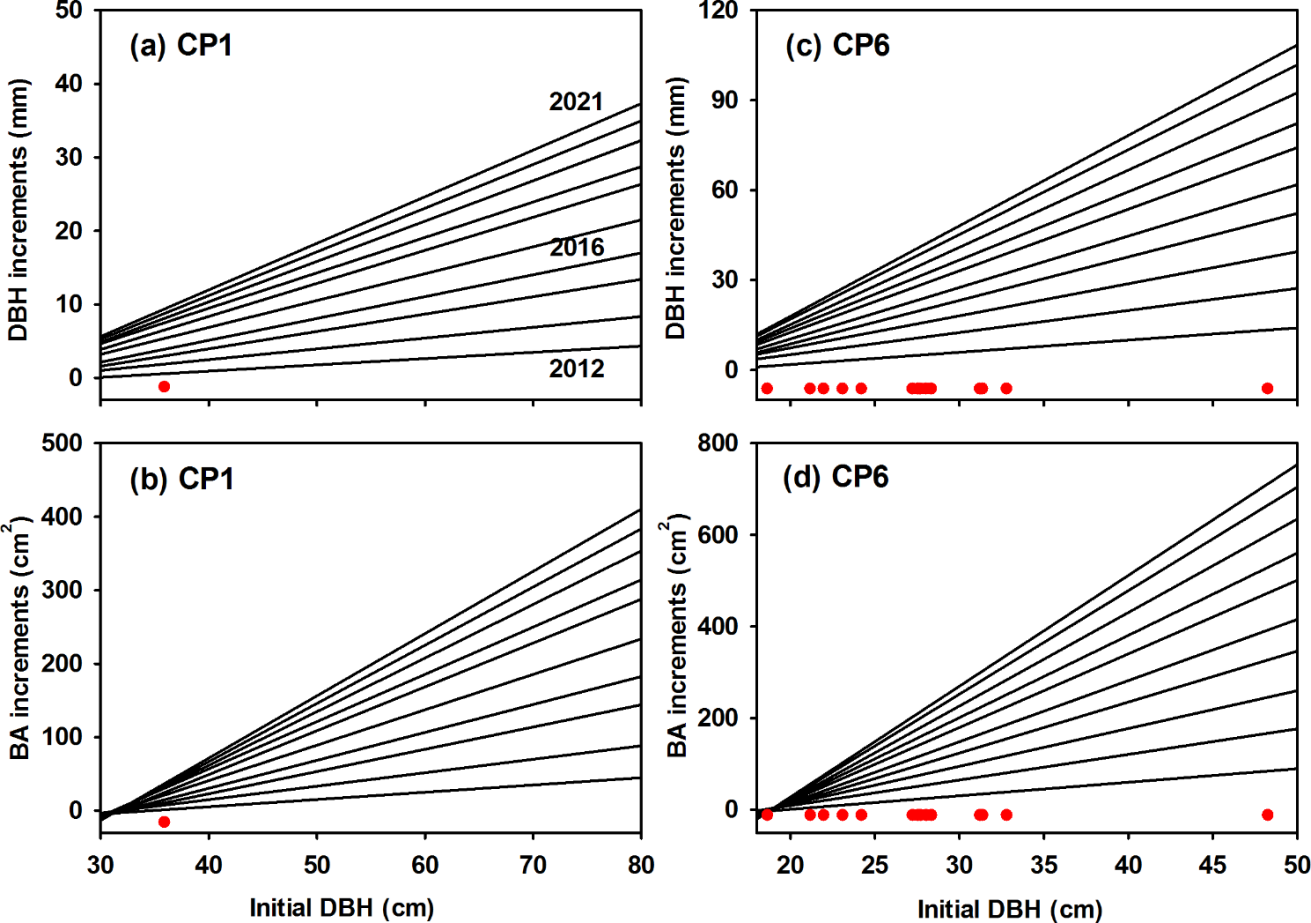
Linear regression between cumulative DBH/BA increments and initial DBH for each year’s measurements from 2012 to 2021 at CP1 and CP6 stands. The red dots represent the initial DBH of the dead trees.

The rates of individual tree growth were correlated with tree size (Fig. 5; Table S1). The initial DBH was a good predictor for predicting DBH and BA (DBH/BA) increments and showed significant linear relationships between cumulative DBH/BA increments and initial DBH for each year’s measurement (Fig. 5 & S4; Table S1). Based on the 10-year measuring periods, the linear regression slope between cumulative DBH/BA increments and initial DBH continued to increase with the years. The intercepted values at the initial DBH, however, did not vary with the years. The smallest trees had minimal DBH/BA increments and showed higher mortalities.

## Discussion

### Homemade steel dendrometer bands

The consistency of the band and tape observations indicate that the homemade stainless steel dendrometer bands are suitable for monitoring the long-term diameter increments of trees. The dendrometer bands used in this study are resistant to high humidity, high temperatures, and mechanical disturbances due to typhoons and heavy rain (Cheng et al. 2020). The results also suggest that the dendrometer bands performed well on both coniferous and broadleaf trees. They were effective for trees with initial DBH of 16.4–71.6 cm for coniferous trees and of 7.0–26.3 cm for broadleaf trees. The measuring range of diameter increment for each tree was from 0 mm to 115.6 mm and 135.4 mm for the coniferous and broadleaf trees, respectively (Table 2).

A comparison of all trees (n = 492) revealed no significant differences between diameter tape and dendrometer bands. The mean differences between two measurements were typically less than 2 mm, but a difference was observed for the CP3 and BR2 stands. The discrepancies may be associated with measurement error. In this study, we painted measurement location on the trunks to minimize the errors from remeasurements. However, high precipitation in Xitou might fade the paint rapidly and grant the mark of some trees unmarkable in the following measurements (Cheng et al. 2020). Other errors, such as neglecting to account for the tension of the tape on trees or to level the tape at the proper plane, may have potentially affected the quality of the results and have caused the systematic differences (Williams et al. 1999). “Missing band length” may also occur to the mechanics of the band dendrometer, where friction may hold the band without slipping back as the stem shrinks, particularly in dry winter when we conducted the annual measurements (Sheil 2003). The lower measuring values from band measurements compared to tape measurements at CP3 and BR2 may thus derive from this reason. Soil water characteristic, tree physiology, and stem friction and deformation at these stands could contribute to the differences. Nevertheless, the mean differences in annual diameter increments between the two measurements were 0.2 mm yr^− 1^ for CP3 and 0.8 mm yr^− 1^ for BR2 (Table 2). The values were acceptable because diameter measurement errors less than ± 0.015 × DBH are tolerated (You et al. 2016).

To the best of our knowledge, our 10-year dendrometer band measurement period is the longest published tree diameter measurement period (Keeland and Shariz 1993; Carvalho and Felfili 2011). The long service life of the dendrometer bands can be attributed to the use of stainless steel. Moreover, the long extension length of the spring provides ample space for long-term monitoring in the field. For examples, two or three years measurement periods and maximum extension length of the spring at around 60 mm were commonly conducted (Keeland and Shariz 1993; Pelissier and Pascal 2000; Pesonen et al. 2004; Drew and Downes 2009; Carvalho and Felfili 2011). In this study, the maximum diameter increments were measured at 115.6 mm and 135.4 mm for coniferous and broadleaf trees, respectively, equaling 363 mm and 425 mm extensions in band circumference.

The dendrometer bands devised in this study were demonstrated to be reliable and durable and are thus suitable for long-term measurements of tree growth. Several managemental features can be highlighted when using the bands in forestry and forest ecology research, including:

(i) Accuracy in repeated measurements

More accurate repeated measurements can be achieved with dendrometer bands than with diameter tape or caliper measurements because the bands are fixed at the same place on the trunk. Figure 4 reveals the bands are able to record annual stem diameter changes at a fine scale resolution, and presents the applications in growth studies. The accuracy of submillimeter precision exceeds many practical needs for evaluating many ecological and silvicultural research questions. In addition, errors in reading the bands can be minimized and measurements are less prone to bias (Cattelino et al. 1986). The required measurement time in the field is also shorter. For large trees, measurements using calipers or diameter tape may take substantially longer than with dendrometer bands (Binot et al. 1995).

(ii) Simple installation and manipulation

The spring-based dendrometer bands used in this study feature straightforward installation. Many band designs require that nails or screws be driven into the tree to support the instrument or serve as a fixed reference against tree growth. Our design does not require screws to be inserted, which is important in long-term studies (Breitsprecher and Hughes 1975). These disturbances may cause tree damage or abnormal growth (Anemeat and Middleton 2013). Installing a dendrometer band takes approximately 5 minutes. Yet, the device can continually be used to monitor the tree growth for at least 10 years without replacing the bands.

(iii) Low cost

Homemade dendrometer bands are considerably more cost-effective than commercially available dendrometer bands. Our stainless steel band and spring design costs US$5 per set. By contrast, commercial dendrometer bands are more expensive, and data collection involves further cost. Commercial bands can be used to make precise measurements with a high time resolution at daily or weekly intervals, but their high prices make them unsuitable for installation on different trees or stands. Homemade dendrometer bands can acquire sufficient spatial data and are commonly employed in studies (Just and Frank 2019).

We purchased the stainless steel bands and springs from a local hardware store in Taiwan. Similar components are also available from online hardware companies (e.g., Misumi USA, WW Grainger, and McMaster-Carr). The easy construction, simple installation, low cost, durability, and long-term reliability of these dendrometer bands make them suitable for monitoring tree growth. We believe these dendrometer bands could be routinely used in forest inventory programs or citizen science projects (Just and Frank 2019). In the future, these dendrometer bands can be improved by implementing automatic recording, along with a wireless transmitter, to achieve a more efficient and temporally accurate device for long-term measurements.

### Applications of dendrometers: examples

Figure 4 and Fig. 5 demonstrate well of using the dendrometer bands for monitoring individual tree growth at a finer scale and clearly show that the trees grew steadily over the past ten years in Xitou. Steady tree growth in Xitou could be due to the weather conditions in which less temperature and precipitation stress permits the individual trees to grow annually. However, further investigation is needed to elucidate how tree growth varies in a single year since the variations in temperature, precipitation, and extreme weather conditions (e.g., typhoon events) influence annual tree growth (Clark et al. 2000). In these even-aged stands, the growth of larger trees was faster than smaller trees and kept the growing paces over ten years (Fig. 6). Similar results have been reported in many studies, in which the growth in DBH/BA is highly dependent on tree size (Masaki et al. 2006; Inoue et al., 2008; Fukumoto et al. 2020). Masaki et al. (2006) concluded that larger trees occupy more leaf mass and have more energy supply for tree growth. By contrast, the growth of smaller trees was suppressed by asymmetric competition from the larger trees and showed higher mortality. Understanding individual tree growth patterns is crucial to develop optimal forest management.

The mean annual diameter increments for the coniferous stands in Xitou were approximately 2–4 mm. These results fall within the range of previous studies (Cheng et al. 2013, 2014). Cheng et al. (2013, 2014) and Lin et al. (2022) reported that the mean annual DBH growth rates of Japanese cedar and *Taiwania* stands were 2.5–4.0 mm in Xitou and northern Taiwan. In Japan, the mean DBH growth rates of Japanese cedar stands ranged from 2.2 to 5.9 mm (Masaki et al. 2006; Hiroshima et al. 2020). The mean annual diameter increments for the broadleaf stands were approximately 5–6 mm, which is consistent with the results of relevant studies in Taiwan (Chan et al. 2015; Cheng et al. 2016).

## Conclusions

We compared the diameter increments measured with dendrometer bands and with diameter tape for 5- and 10-year measurement periods. The results indicated that the two measurements were highly correlated (R > 0.89). The differences between the two measurements of the individual trees were typically less than ± 5 mm, and the mean differences at the stand level were less than ± 2 mm. Overall, the data (n = 492 for the 10-year measurement period on both coniferous and broadleaf trees) were not significantly different between the two measurements (*p* = 0.16). The dendrometer bands developed in this study were reliable, durable, and suitable for long-term measurements of tree growth. The bands have several features beneficial for forestry and forest ecology research, including (i) accuracy across repeated measurements, (ii) simple installation and manipulation, and (iii) low cost. Automatic data recording could improve these dendrometer bands to achieve a more efficient and temporally accurate device for long-term measurements. However, the automatic recording system should be considered for power supply and security of the instrument because the experimental sites are usually locate in outlying areas.

## Electronic supplementary material

Below is the link to the electronic supplementary material.

Additional File 1: Relationship between the spring length and the force produced by the spring. Photos of the materials and tools for making the homemade stainless steel dendrometers. Installation and measurement of the dendrometer bands in the field. Examples of the relationships and related parameters between cumulative DBH/BA increments and initial DBH.

## Data Availability

The data used to support the findings of this study are available from the authors upon request.
