# Supplementary material for: Using homemade stainless steel dendrometer band for long term tree growth measurements
Source: Bot Stud. 2023 Jul 19;64:22. doi: 10.1186/s40529-023-00395-8 (PMC10356724; doi:10.1186/s40529-023-00395-8)
Supplement: Supplementary file 1 — Additional File 1: Relationship between the spring length and the force produced by the spring. Photos of the materials and tools for making the homemade stainless steel dendrometers. Installation and measurement of the dendrometer bands in the field. Examples of the relationships and related parameters between cumulative DBH/BA increments and initial DBH. [file 40529_2023_395_MOESM1_ESM.docx]

**Supplementary files**

Figure S1. Relationship between the spring length and the force produced by the spring. The spring has a wire diameter of 0.8 mm, an outside diameter of 8 mm, a length of 150 mm, and 187 coils. The maximum extension length and force are 44.8 cm and 25 N, respectively.

Figure S2. Materials and tools used for making the homemade stainless steel dendrometers. (a) Stainless steel spring, (b) stainless steel band, (c) metal snip, (d) hammer, (e) wire brush, (f) gloves, and (g) concrete nail.


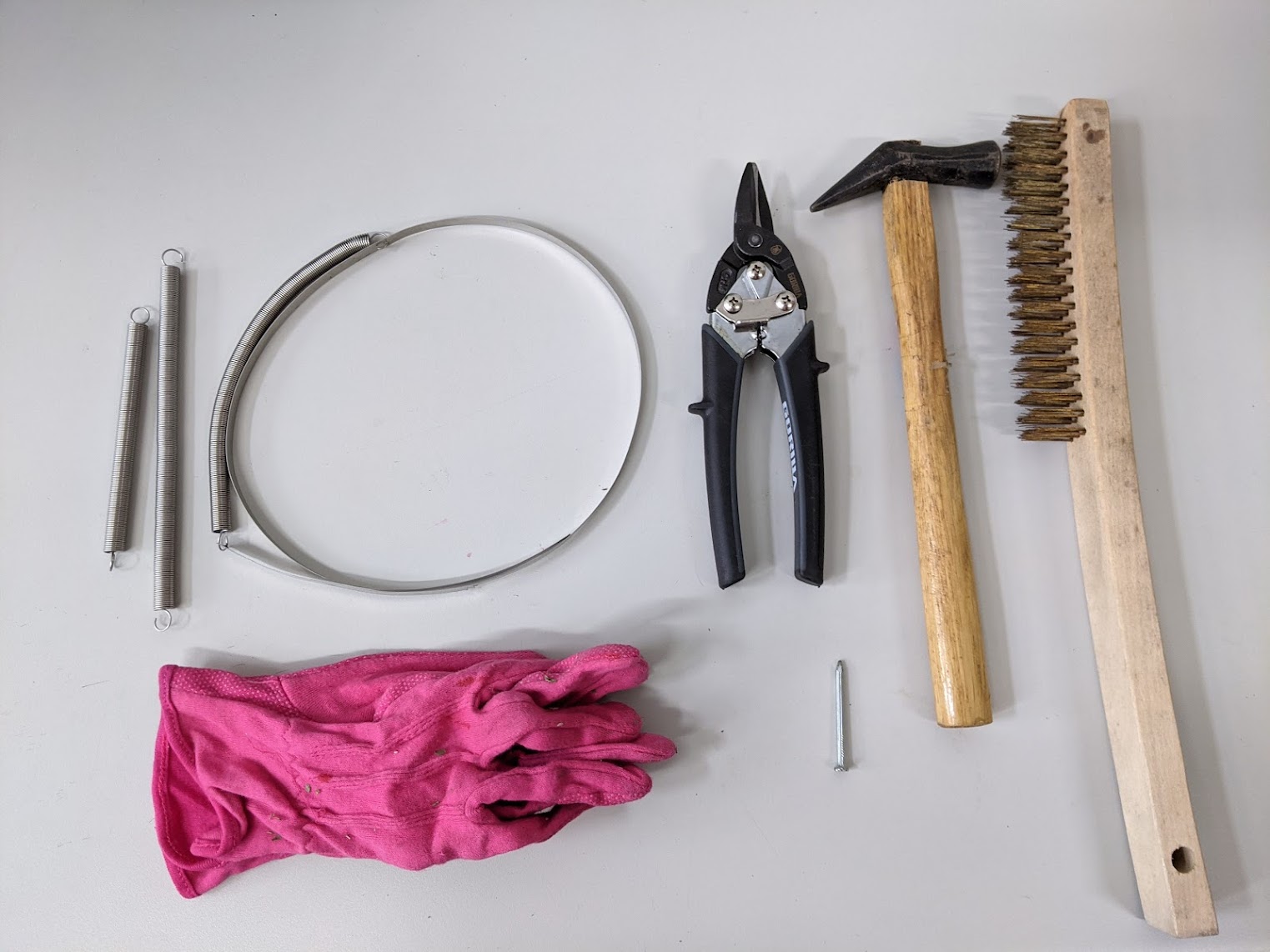


**(e)**

**(g)**

**(f)**

**(d)**

**(c)**

**(a)**

**(b)**

Figure S3. Installation and measurement of the dendrometer bands in the field. (a) Photos of a dendrometer band in the field. (b) Hole at the end of the band with the spring connected through the hole. (c) Second hole for extending the other end of spring to connect through which permitting the band a tight fit on the trunk. (d) Tree growth refers to the distance from notch to the moving end of the band.

Figure S4. Examples of the relationships between cumulative DBH/BA increments and initial DBH in the year of 2012, 2016 and 2021 at CP1 and CP6 stands. The solid circles are the measurements of individual trees in 2012, empty circles in 2016, and triangles in 2021. The black lines are the linear regression in each year.

Table S1. Parameters of linear regression between cumulative DBH/BA increments (mm/cm^2^, y) and initial DBH (cm, x) for each year’s measurements from 2012 to 2021 at CR1 and CR6 stands. Significant relationships were found for each regression line.

|  | DBH increments  (mm) | R^2^ | BA increments  (cm^2^) | R^2^ |
| --- | --- | --- | --- | --- |
| **CR1** |  |  |  |  |
| 2012 | y=-2.484+0.085x | 0.243 | y = -34.12+0.987x | 0.343 |
| 2013 | y=-3.367+0.146x | 0.246 | y = -58.02+1.829x | 0.394 |
| 2014 | y=-5.502+0.236x | 0.297 | y = -98.18+3.029x | 0.460 |
| 2015 | y=-6.756+0.297x | 0.267 | y = -121.4+3.800x | 0.424 |
| 2016 | y=-7.745+0.366x | 0.263 | y = -151.8+4.816x | 0.432 |
| 2017 | y=-9.633+0.450x | 0.268 | y = -189.5+5.970x | 0.437 |
| 2018 | y=-9760+0.481x | 0.264 | y = -200.6+6.437x | 0.438 |
| 2019 | y=-11.60+0.549x | 0.258 | y = -230.1+7.299x | 0.423 |
| 2020 | y=-12.58+0.595x | 0.258 | y = -250.0+7.922x | 0.424 |
| 2021 | y=-13.37+0.634x | 0.262 | y = -267.4+8.474x | 0.431 |
| **CR6** |  |  |  |  |
| 2012 | y=-6.307+0.407x | 0.309 | y = -57.70+2.948x | 0.472 |
| 2013 | y=-9.588+0.737x | 0.364 | y = -102.5+5.590x | 0.565 |
| 2014 | y=-14.06+1.071x | 0.379 | y = -152.0+8.238x | 0.578 |
| 2015 | y=-20.33+1.452x | 0.397 | y = -209.7+11.13x | 0.574 |
| 2016 | y=-23.90+1.715x | 0.396 | y = -251.8+13.34x | 0.583 |
| 2017 | y=-28.60+2.056x | 0.421 | y = -302.5+16.08x | 0.610 |
| 2018 | y=-31.70+2.280x | 0.411 | y = -337.8+17.97x | 0.596 |
| 2019 | y=-36.36+2.578x | 0.412 | y = -385.9+20.42x | 0.592 |
| 2020 | y=-39.58+2.828x | 0.398 | y = -425.4+22.60x | 0.576 |
| 2021 | y=-42.42+3.018x | 0.394 | y = -455.6+24.19x | 0.568 |
